# Thermal decomposition characteristics of BHT and its peroxide (BHTOOH)

**DOI:** 10.1186/s13065-024-01190-7

**Published:** 2024-04-29

**Authors:** Suyi Dai, Min Liang, Haijun Cheng, Chang Yu, Weiguang Li, Fang Lai, Li Ma, Xiongmin Liu

**Affiliations:** 1 School of Chemistry and Chemical Engineering, Guangxi University, Nanning, 530004 Guangxi China; 2 Department of Chemistry, Hengshui University, Hengshui, 053000 China

**Keywords:** Thermal stability, Decomposition properties, The mini closed pressure vessel test (MCPVT), Safety of chemical industry

## Abstract

2,6-Di-tert-butyl-4-methylphenol (BHT) is an excellent antioxidant that is easily oxidized to 2,6-di-tert-butyl-4-hydroperoxyl-4-methyl-2,5-cyclohexadienone (BHTOOH). For the safety of BHT production and usage, it is meaningful to study the thermal stability and decomposition properties of BHT and BHTOOH. In this paper, the thermal decomposition properties of BHT and BHTOOH were compared by the mini closed pressure vessel test (MCPVT) and differential scanning calorimetry (DSC). Their kinetics of thermal decomposition were studied using thermogravimetric analysis (TGA). The thermal decomposition products of BHT and BHTOOH were analyzed by gas chromatography-mass spectrometry (GC–MS). The results show that there was no significant change in temperature pressure when BHT was warmed up under a nitrogen atmosphere, indicating that BHT was stable within 400 K. The thermal decomposition reaction of BHTOOH was rapid with an initial reaction temperature of 375.2 K. The initial exothermic temperature (T_i_) and heat release (Q_DSC_) of DSC were 384.9 K and 865.0 J g^−1^, respectively. The apparent activation energies (E_a_) for the thermal decomposition reactions of BHT and BHTOOH calculated by the Kissinger method were 151.8 kJ mol^−1^ and 66.07 kJ mol^−1^, respectively. The main decomposition products of BHT were isobutene and 2-tert-butyl-4-methylphenol. The thermal decomposition products of BHTOOH included BHT, 2,6-di-tert-butyl-4-ethylphenol, 3,5-di-tert-butyl-4-hydroxybenzaldehyde, 4,4′-(1,2-ethanediyl) bis [2,6-bis (1,1-dimethylethyl) phenol, etc. Based on the thermal decomposition behavior and products, the reaction pathway has been described. These results indicate that BHT is a potential thermal hazard during production, storage and application. For the safety of the chemical industry, the oxidation of BHT should be avoided.

## Introduction

2,6-Di-tert-butyl-4-methylphenol (BHT) is one of the most prevalent synthetic phenolic antioxidants. In 2000, it was estimated that BHT was distributed in various fields such as rubber (27%), plastics (27%), mineral oil/fuel additives (17%), and food/drugs/cosmetics (12%) [1]. Due to the wide application of BHT, it can reach an annual production of 9000 tons in China [2]. The mass production and widespread use of BHT has resulted in its exposure to the environment as well as the human body [3–7]. 2,6-Di-tert-butyl-4-methyl-4-hydroperoxy-2,5-cyclohexadienone (BHTOOH) is one of the toxic metabolites of BHT. Much attention has been given to the toxicity of BHTOOH. Yamamoto K. et al. studied the acute toxicity of BHT and four metabolites. The results showed that BHTOOH was probably the most toxic metabolite, with an i.p. LD_50_ value of 190 mg kg^−1^ [8]. The metabolite of BHT, BHTOOH, has been reported to cause DNA strand damage[9]. Furthermore, BHTOOH is also a promoter of skin tumors in mice and can cause toxicity in the liver and lungs [10].

BHTOOH is an oxidation product of BHT, and the reaction is easy to carry out [11]. BHT is often added to foods such as cooking oils, fats, and crackers, which encounter high temperatures during cooking [12]. BHT may react with oxygen during this process to form BHTOOH. BHT is also added to plastics. Mineral water bottle caps undergo oxidative degradation under light and heat conditions. The additive BHT and its transformations in bottle caps can migrate into drinking water [13]. This process involves oxidative degradation of BHT. BHT is also a thermal oxidation stabilizer for jet fuel. If high concentrations of BHT are added to fuel, more severe thermal deposition occurs due to the generation of phenoxy radical [14–16]. In these applications, oxidation of BHT may result in the formation of the organic peroxide BHTOOH. Organic peroxides are very unstable and have exothermic hazards [17]. Once BHT is oxidized to form BHTOOH during transport, storage and use, a potential thermal hazard arises. Therefore, the potential thermal hazards of BHT cannot be ignored.

It is important to study the thermal stability and thermal decomposition properties of BHT and BHTOOH. There are relatively few studies on the thermal properties of BHT and BHTOOH. Fujisawa S used the induction period method with differential scanning calorimetry (DSC) to investigate the radical-scavenging activity of BHT and its metabolites in the polymerization of methyl methacrylate (MMA) initiated by thermal decomposition of AIBN or BPO. It was found that BHT was effective as a chain-breaking antioxidant [18]. At frying temperature, BHT was the least stable compared to BHA, TBHQ, and PG, in part because decomposition occurs [19]. Warner et al. investigated the chemical transformations of BHT and BHA during potato frying and found that BHT could be cleaved to BHT-CHO, BHT-OH and BHT-Q. The obvious oxidation product of BHA and TBHQ was TBBQ [20]. Buben and Pospíšil studied the behavior of several alkyl peroxycyclohexadienones (including BHTOOH) using DSC and TG. It was found that several selected peroxycyclohexadienyl alkyketones showed characteristic characteristics in DSC tests, that is, melting to absorb heat and then release heat [21]. It can be found that the current research on the thermal characteristics of BHT and BHTOOH is not deep enough, and the specific parameters such as decomposition temperature, the rate of heat release and quantity of heat need further research.

To study the thermal stability of BHT and BHTOOH, the mini closed pressure vessel test (MCPVT) was used to measure the temperature and pressure of during their heating process. The thermal decomposition characteristics of BHT and BHTOOH were determined by DSC and their hazards were evaluated. Their thermal decomposition kinetics were studied by TG method. The thermal decomposition products were collected and analyzed using GC–MS. Furthermore, we described the BHTOOH thermal decomposition reaction pathway. This study is valuable for understanding the thermal decomposition characteristics and hazards of BHT and BHTOOH, and provides a reference for the safe production, storage and application of BHT.

## Materials and methods

### Materials

BHT (99.00%) was supplied by Shanghai Macklin Biochemical Co., Ltd. KOH (99.50%), acetic acid (99.50%), and n-hexane (97.00%) were purchased from Guangdong Guanghua Sci-Tech Co., Ltd. KBr (99.99%) was provided by Tianjin Guangfu Fine Chemical Research Institute. N_2_ and O_2_ (99.99%) gases were purchased from Nanning Yunlaida Gas Co., Ltd, China.

### Preparation of BHTOOH

BHTOOH was formed by the oxidation of BHT [22]. The specific steps were as follows: BHT (4.4 g) was solubilized in ethanol (50 mL), and potassium hydroxide solution (2 g in 5 mL water) was then added. The solution was passed through the flask with 450 mL of oxygen and vigorously stirred for approximately 30 min. After absorbing (0.02 mol) oxygen, the solution that turned pale yellow was instantly poured into ice water (700 mL) and neutralized with acetic acid. The separated sediment was gathered on a filter, washed with water, and dried. Crystallization from n-hexane gave colorless needles (BHTOOH), melting at 388–389 K.

### Thermal stability of BHT and BHTOOH using MCPVT

**Fig. 1:**
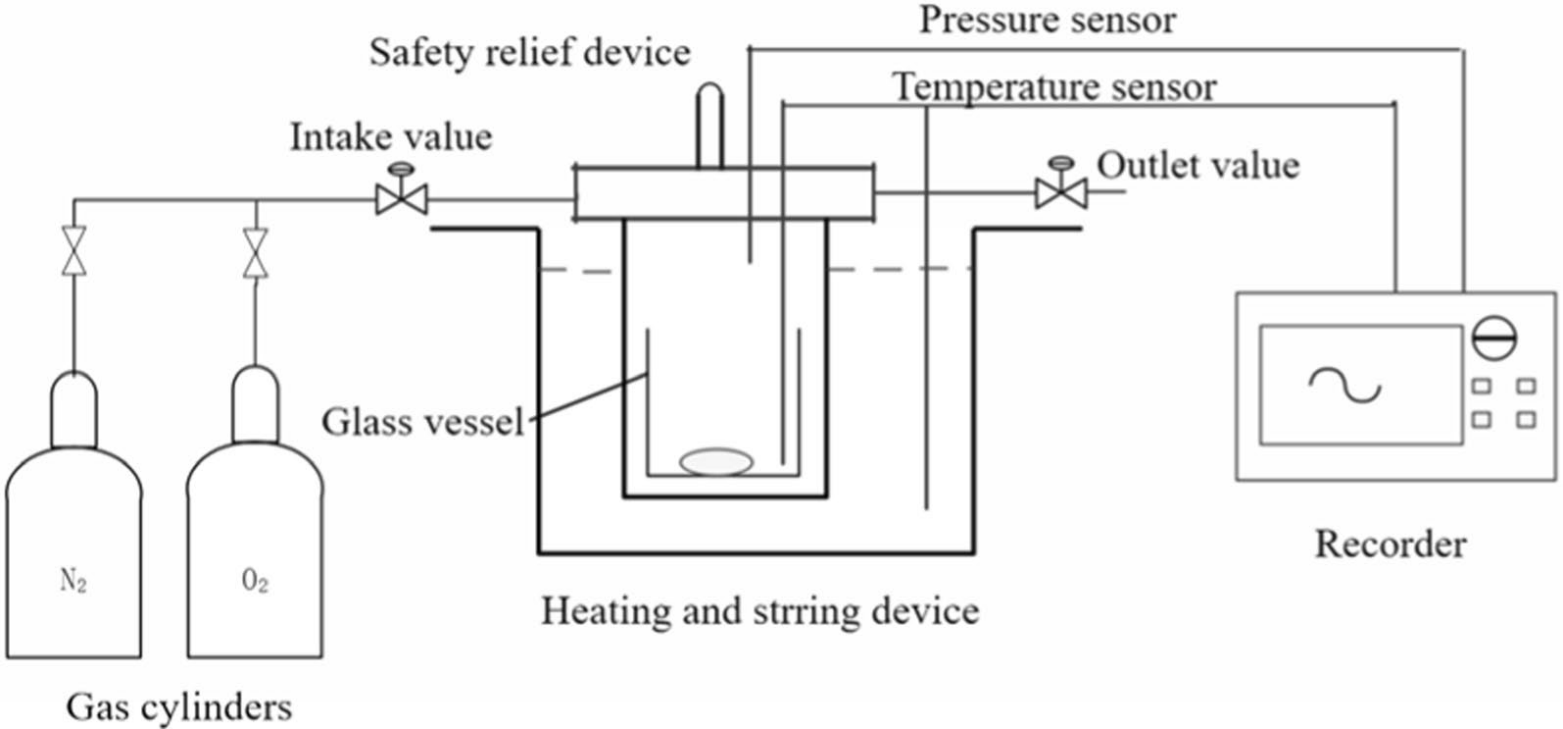
Experimental apparatus for BHTOOH oxidation reaction

MCPVT was applied to trace the thermal decomposition reaction of BHT and BHTOOH. The experimental setup is shown in Fig. 1. The reactor used in the experiment is a mini-closed pressure vessel made of stainless steel. The temperature sensor, pressure sensor and memory recorder are manufactured in Japan with models CC-3083, cd-700a and 8860-50 respectively [23]. The specific experimental procedure was as follows: A glass test tube containing 0.3 g of BHTOOH was placed in a mini-closed pressure vessel with a capacity of 35 mL. The closed pressure vessel was filled with nitrogen (0.4 MPa), oxygen (0.4 MPa), and air (0.1 MPa; 0.4 MPa). As a comparison, BHT (1.56 g) was also placed in a glass test tube under a nitrogen atmosphere of 0.4 MPa. The temperature rose from room temperature to 418.3 K.

### Thermal decomposition properties of BHT and BHTOOH

The thermal hazard of BHT and BHTOOH was determined by DSC, a convenient instrument for assessing the hazards of organic peroxides [24]. Dynamic temperature programmed screening experiments were performed on a Q2000 TA DSC instrument. BHT and BHTOOH (about 4 mg) were placed in a sealable test crucible, respectively. Dynamic scanning tests were performed in the range of 303 to 473 K at a specific heating rate (10 K min^−1^) under a nitrogen atmosphere. The detection sensitivity was 0.2 μW.

TG experiments were performed with a NETZSCH STA 2500 instrument under a nitrogen atmosphere at a flow rate of 50 mL min^−1^. The crucible was made of alumina and contained approximately 6.0 mg BHTOOH. The heating rates were 5, 10, 15, 20, and 25 K min^−1^, and the temperature was increased from 303 to 473 K. As a comparison, TG tests were performed on BHT at 300–700 K. 

### Analysis of thermal decomposition products of BHT and BHTOOH

The peroxide thermal decomposition process results in the formation of many complex products accompanied by the formation of small molecules. The liquid and gaseous products of the decomposition of BHT and BHTOOH were measured as GC/MS-QP2010 (Shimadzu, Japan) with a built-in Rxi-5Sil fused silica capillary column (30 m × 0.25 mm × 0.25 μm) coupled with an electron impact ionization detector (70 eV). The GC temperature program was as follows: the initial temperature was 373 K. Then the temperature was ramped up to 513 K at a rate of 15 K min^−1^ and maintained for 3 min. The injection temperature and volume of the samples were 533 K and 1.0 µL, respectively, with a split ratio of 80:1. The interface temperature was 523 K, the ion source temperature was 473 K, and the scan mass range m/z was 40–500.

## Results and discussion

### Synthesis and structural characterization of BHTOOH

**Fig. 2:**
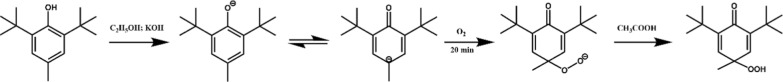
Synthetic route of BHTOOH

BHTOOH was obtained by a base-catalyzed reaction. The synthesis route of BHTOOH (Fig. 2) was as follows: KOH removed the hydrogen from the phenolic hydroxyl group on BHT to form the phenoxyanion. Then it reacted with oxygen to produce the peroxide anion of the cyclohexadienone structure, which was finally neutralized by acetic acid to produce BHTOOH. The obtained transparent needles were structurally characterized by high-resolution mass spectrometry (HRMS), ^1^H and ^13^C nuclear magnetic resonance (NMR), and Fourier transform infrared spectroscopy (FTIR).

**Fig. 3:**
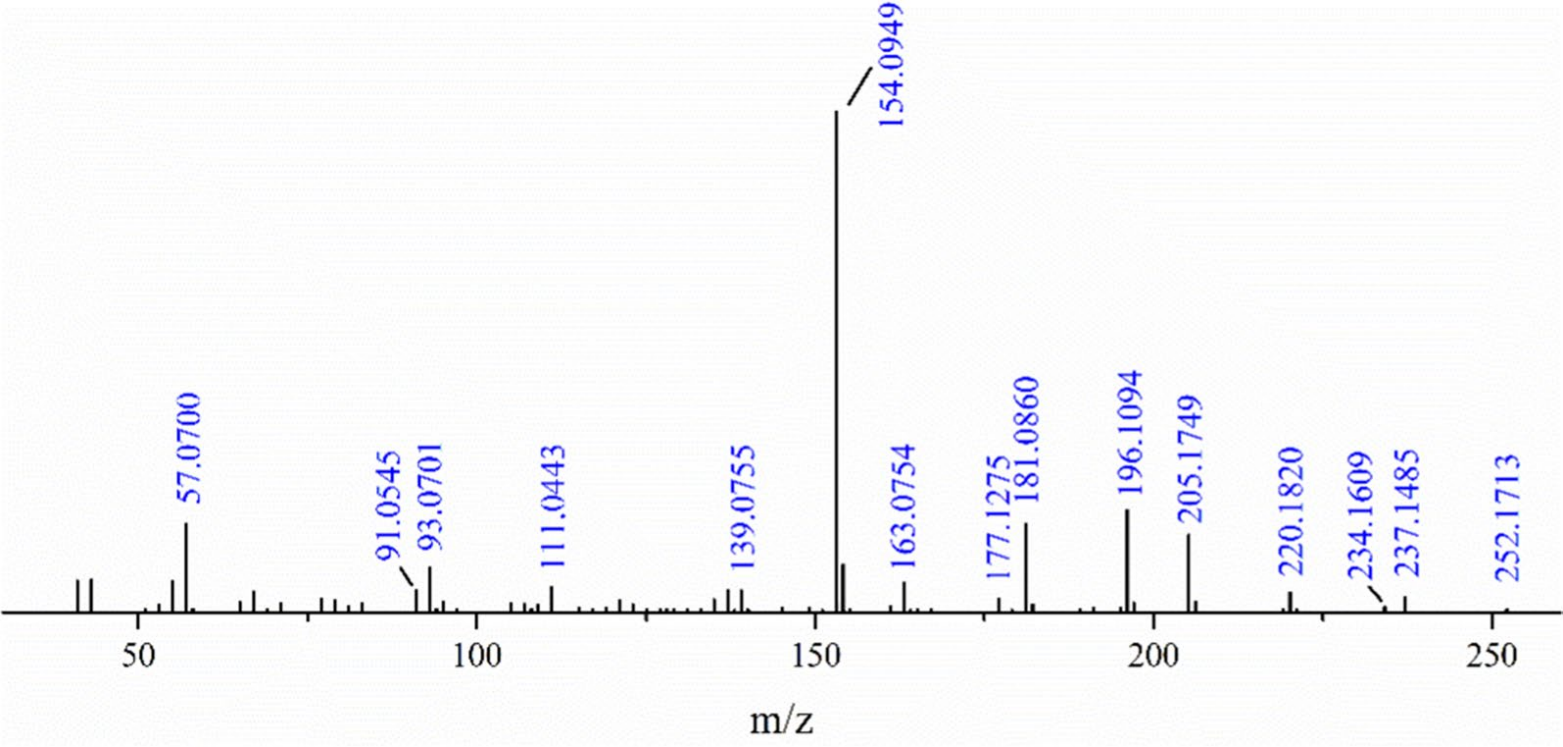
Mass spectrum of BHTOOH

The mass spectra of BHTOOH were determined by HRMS (Agilent Technologies 7250 GCQTOF) with electron ionization (EI) running in the negative-ionization mode. The HRMS results represented signals at m/z 252.1713, 237.1485, 220.1820, 196.1054, 154.0949, and 57.0700, as shown in Fig. 3. The signal at m/z 252.1713 represented the M^+^ molecular ion peak of BHTOOH with an increase in molecular weight from 220.1820 to 252.1713, which should be attributed to the combination of BHT and oxygen.

**Fig. 4:**
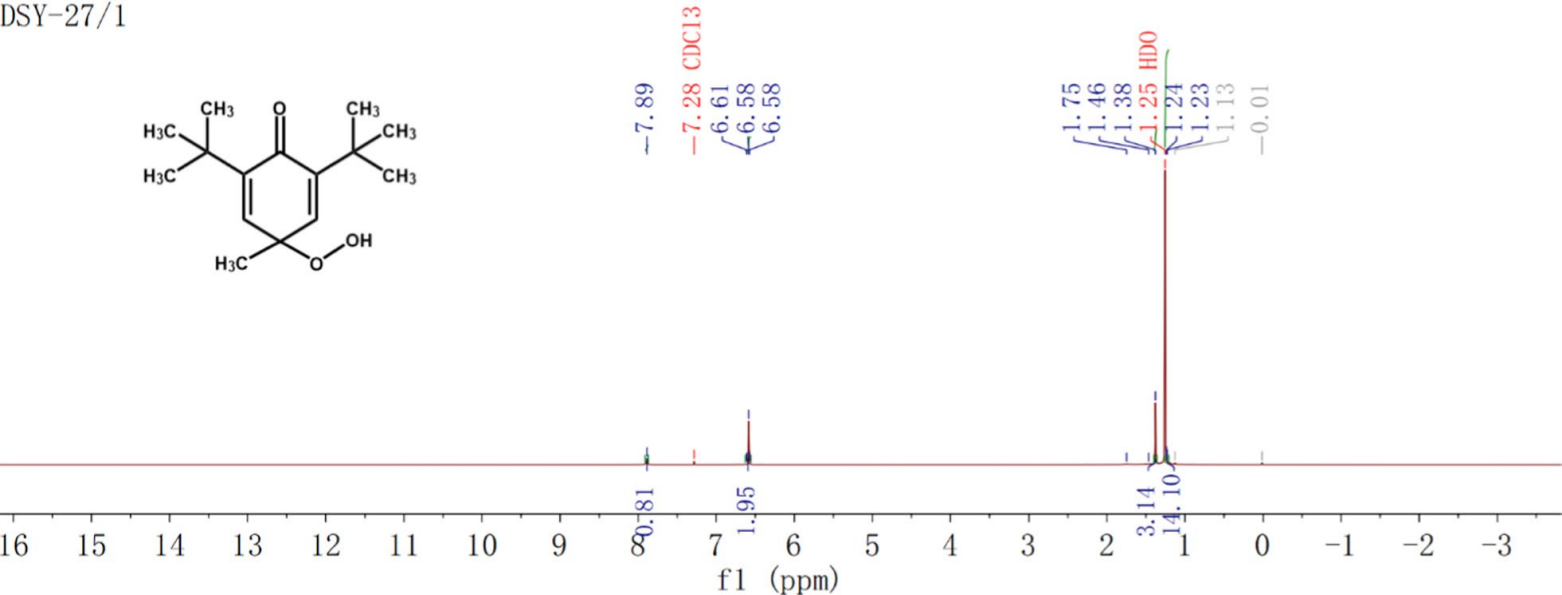
^1^H NMR spectra of BHTOOH

**Fig. 5:**
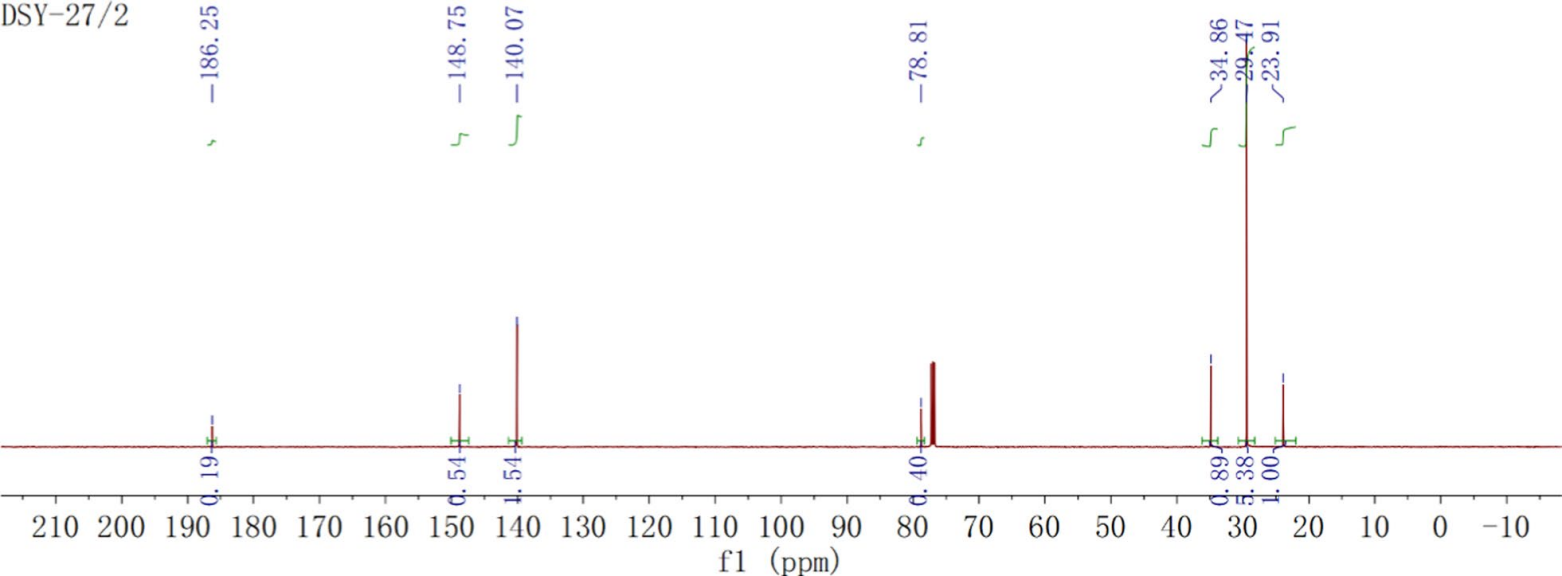
^13^C NMR spectra of BHTOOH

To verify the structure of BHTOOH, further structural characterizations were performed by ^1^H and ^13^C NMR (Fig. 4 and Fig. 5). Approximately 20 mg of BHTOOH was dissolved in 0.5 mL of CDCl_3_ and charged into an NMR tube for measurement by the NMR test (AVANCE III HD 600 spectrometer, Bruker, Switzerland).

The results were as follows: ^1^H NMR (500 MHz, Chloroform-d): δ 7.87 (s, 1H), 6.56 (s, 2H), 1.36 (s, 3H), 1.23 (s, 18H) and ^13^C NMR (126 MHz, Chloroform-d): δ 186.25, 148.75, 140.07, 78.81, 34.86, 29.47, and 23.91.

**Fig. 6:**
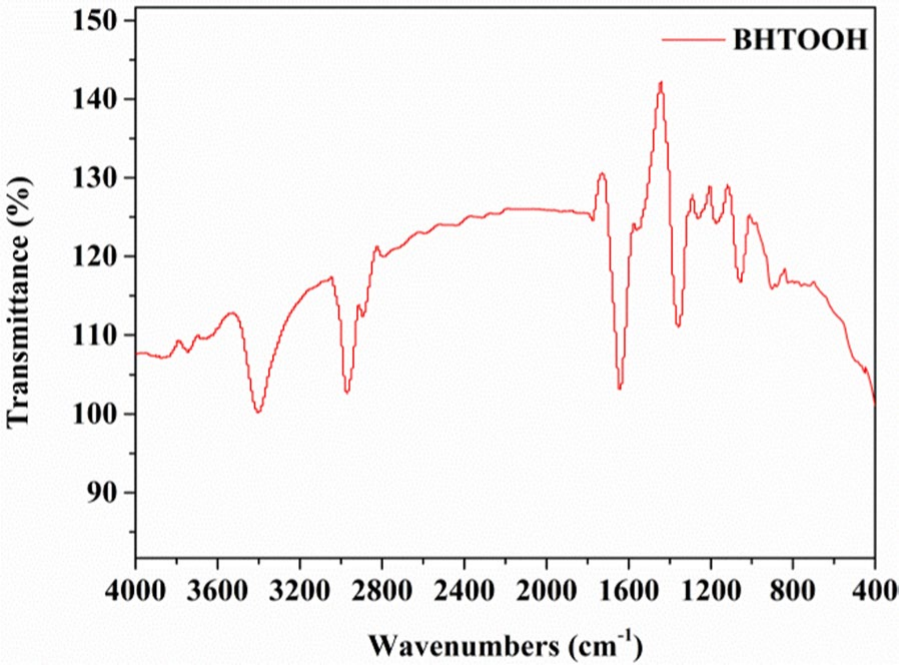
FTIR spectrum of BHTOOH

To identify the functional groups of BHTOOH, FTIR was carried out with a Thermo Trace 1310-Nicolet IS50 spectrometer (THERMO FISHER). The FTIR resolution and the number of scans were set to 4 cm^−1^ and 40, respectively. FTIR spectra were recorded in the range of 4000–400 cm^−1^. BHTOOH was ground with KBr powder and then compressed into a tablet for FTIR testing. Figure 6 shows the FTIR spectrum of BHTOOH. The characteristic peaks of BHTOOH were at approximately 3410, 2980, 1640, 1370, 1070, and 890 cm^−1^. The peaks at 3410, 2980, and 1640 cm^−1^ were caused by the stretching vibrations of the O–H, –CH_3_, and C=O bonds, respectively. The infrared absorption peak at 1370 cm^−1^ was due to the stretching vibration of the tert-butyl group. In addition, the bending vibration at 1070 cm^−1^ and the stretching vibration at 890 cm^−1^ represented the C–O bond and O–O bond, respectively. In summary, the structure of BHTOOH is shown in Table 1.

**Table 1:** Molecular structure of BHTOOH

Name	Formula	Structure	Mass
2,6-Ditert-butyl-4-hydroperoxy-4-methylcyclohexa-2,5-dien-1-one	C_15_H_24_O_3_	〹	252.1713

### Thermal stability of BHT and BHTOOH

**Fig. 7:**
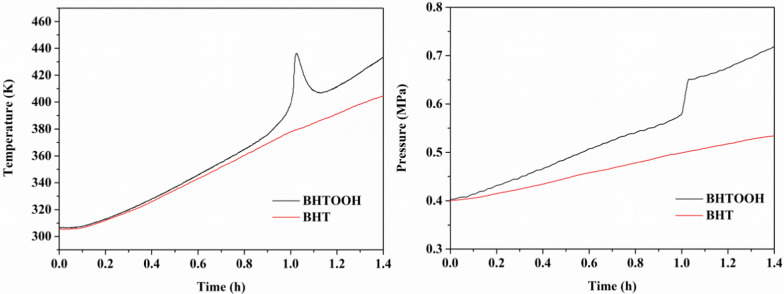
Plots of T–t and P–t for BHT and BHTOOH

MCPVT is commonly used to assess the explosion hazard of organic compounds, especially peroxides. The pressure and rate of pressure rise data obtained from the test are good indicators of the explosion characteristics. The test provides criteria for the risk of exothermicity and deflagration of hazardous materials in a confined environment [25, 26]. BHTOOH is a peroxide of BHT. MCPVT can provide temperature and pressure data for BHT and BHTOOH to model their thermal stability in confined spaces. The temperature versus time (T–t) and pressure versus time (P–t) plots of BHT and BHTOOH are shown in Fig. 7.

Figure 7 shows the temperature and pressure variation of BHT and BHTOOH under a nitrogen atmosphere. The results showed no considerable heat release of BHT during heating. The pressure did not increase rapidly, indicating that BHT is stable. The temperature and pressure of BHTOOH increased rapidly. The increase in temperature is due to the heat generated by the reaction. The rapid increase in pressure indicates that the decomposition of BHTOOH produces a large amount of gas. BHTOOH is unstable and poses a risk of combustion and explosion. Therefore, BHT should avoid contact with oxygen during transportation, storage and use, otherwise BHTOOH may be generated resulting in a hazard.

**Fig. 8:**
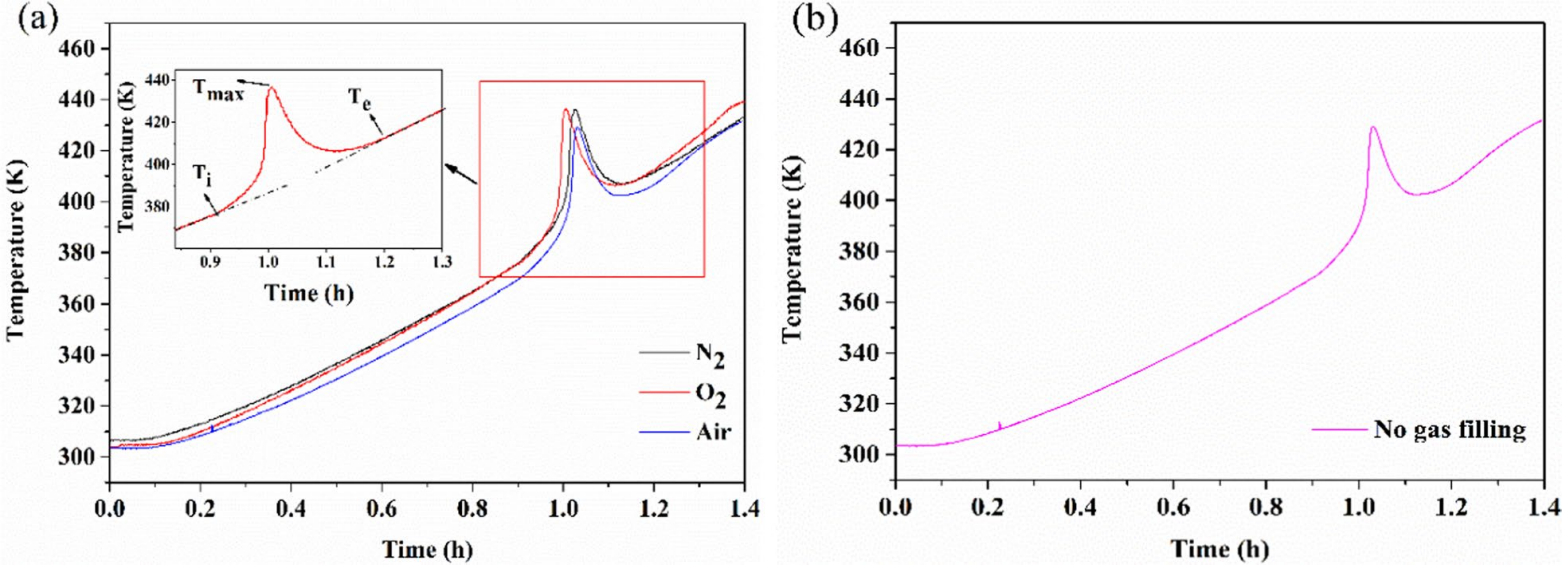
Temperature and time behavior of BHTOOH. **a** Under nitrogen, oxygen, and air atmospheres. **b** Without the addition of a gas atmosphere

**Fig. 9:**
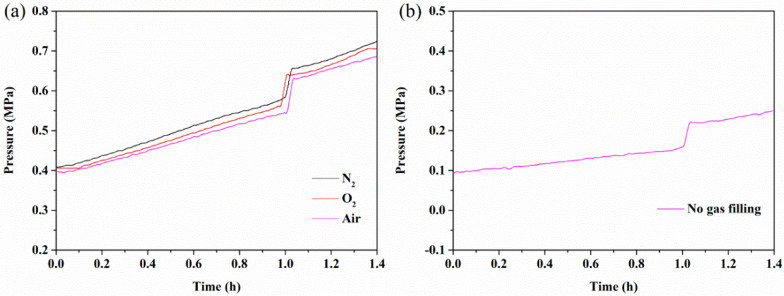
Pressure and time behavior of BHTOOH. **a** Under nitrogen, oxygen, and air atmospheres. **b** Without the addition of a gas atmosphere

To explore the thermal decomposition properties of BHTOOH in depth, the temperature and pressure behaviors of BHTOOH under different gas atmospheres (nitrogen, oxygen, air, and without gas charge) are shown in Fig. 8 and Fig. 9, respectively.

Similarly, the T–t curves all show sharp exothermic peaks under different atmospheres, as shown in Fig. 8a. This indicates that BHTOOH is unstable in different environments and rapidly decomposes when heated. Figure 9a shows that BHTOOH exhibited a rapid increase in pressure under all the different gas atmospheres, and the rise in pressure was expressed as ΔP. The results show that ΔP was positively correlated with the initial pressure of filling. As seen in Fig. 8b and Fig. 9b, BHTOOH underwent thermal decomposition even at atmospheric pressure when heated. BHTOOH is undoubtedly dangerous in a high-temperature confined environment. In addition, the initial exothermic temperature (T_i_) is an essential parameter for assessing the explosion risk of chemicals. The maximum exothermic temperature (T_max_) and the end of exothermic temperature (T_e_) are also vital parameters for understanding the thermal decomposition properties. These critical parameters are listed in Table 2.

**Table 2:** Related parameters of the thermal decomposition of BHTOOH

Gas	P_0_ (MPa)	T_i_ (K)	T_max_ (K)	T_e_ (K)	ΔP (MPa)
N_2_	0.4	375.2	436.3	409.9	0.0731
O_2_	0.4	376.8	436.5	409.0	0.0818
Air	0.4	375.7	426.0	407.4	0.0899
No gas filling	0.1	370.5	429.2	407.0	0.0615

As seen from Table 2, the T_i_ values under the three gas atmospheres were approximately 376 K for the initial gas pressure P_0_ of 0.4 MPa. For P_0_ of 0.1 MPa, T_i_ = 370.5 K. This indicates that the ambient pressure influences the initial reaction temperature of BHTOOH. Correspondingly, ΔP was relatively small at atmospheric pressure. This suggests that the thermal decomposition parameters of BHTOOH are related to temperature and pressure. BHTOOH is more dangerous in high-temperature and high-pressure environments. The results of the MCPVT suggest that BHT should be stored and transported in a low-temperature, low-pressure environment and isolated from oxygen. Otherwise, BHT may be oxidized to form the peroxide BHTOOH, the thermal decomposition of which would cause fire or explosion hazard.

### Exothermic characteristics and hazards of BHT and BHTOOH

**Fig. 10:**
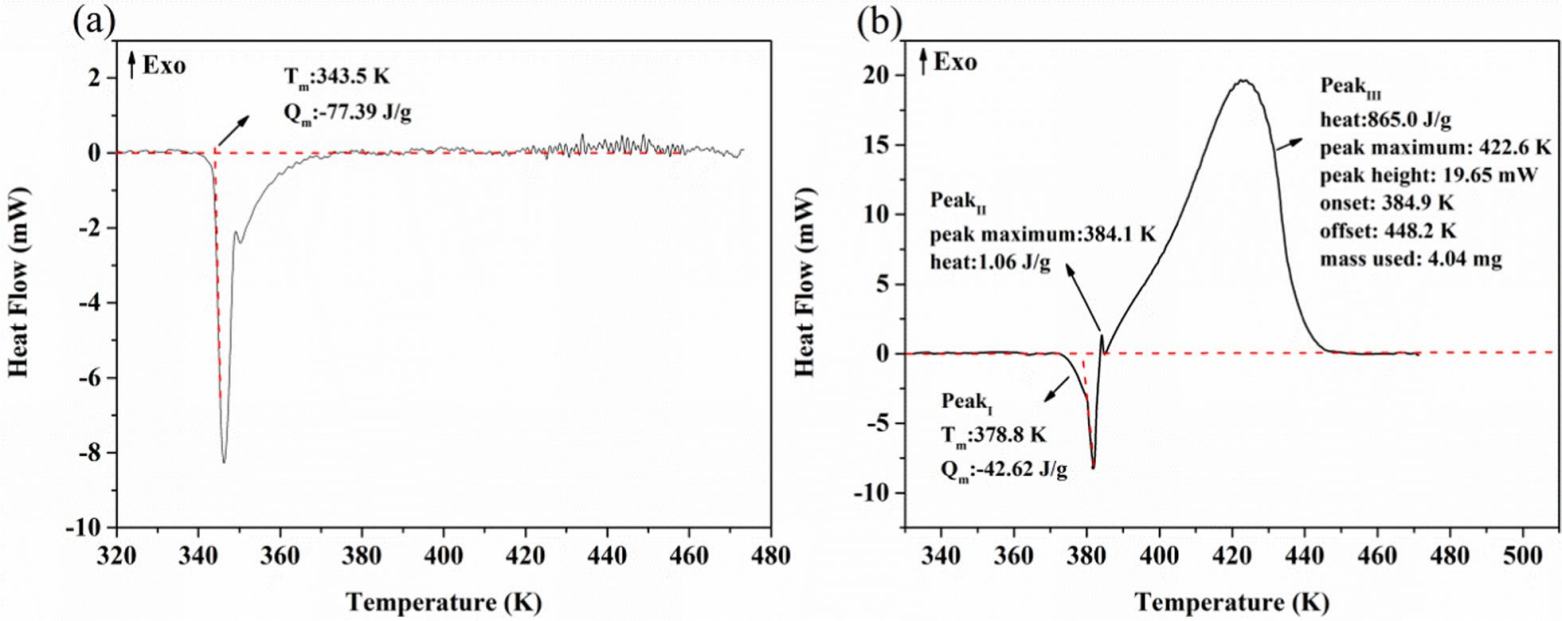
Heat flow curves of BHT and BHTOOH. **a** BHT; **b** BHTOOH

The decomposition of organic peroxides is caused by the breaking of the O–O bond, which generates a large amount of heat and free radicals. A large number of free radicals can trigger explosive polymerization reactions in the material. Due to the high heat of thermal decomposition and the low exothermic onset temperature, organic peroxides can be used directly as potential explosives [27]. DSC is a common means of assessing the hazards of hazardous materials. It provides a reference for the classification of hazardous materials and accident reduction [28]. To observe the thermal hazards of BHT and BHTOOH, a DSC experiment (Fig. 10) is necessary to reveal the thermal properties of BHT and BHTOOH.

DSC testing of BHT and BHTOOH was performed at a ramp-up rate of 10 K min^−1^ from 303.0 K to 473.0 K. Figure 10a shows that BHT (3.67 mg) melted with a melting point (T_m_) of 343.5 K and absorbed heat of 77.39 J g^−1^ (Q_m_). There was no exotherm of BHT during the heating process. However, two exothermic phenomena (Peak_II_ and Peak_III_) occurred immediately after melting (Peak_I_) of BHTOOH, as seen in Fig. 10b, where the second exotherm (Peak_III_) was the primary thermal decomposition process. The results indicate that BHTOOH underwent thermal decomposition and was quite unstable.

The melting point (T_m_) of BHTOOH was 378.8 K, which absorbed 42.62 J g^−1^. The T_m_ was lower than that determined with the b-tube, which may be due to the higher rate of temperature rise. It is noteworthy that BHTOOH decomposed immediately after melting. First, a minor exotherm (Q_DSC_ = 1.06 J g^−1^) occurred at 383.7–384.9 K, followed by a significant heat release (Q_DSC_ = 865.0 J g^−1^) at 384.9 K–448.2 K. This situation was similar to the thermal decomposition of diphenylglyoxime in that both have two rapid exothermic stages after melting, with the second stage being the more dominant exothermic stage [29].

**Fig. 11:**
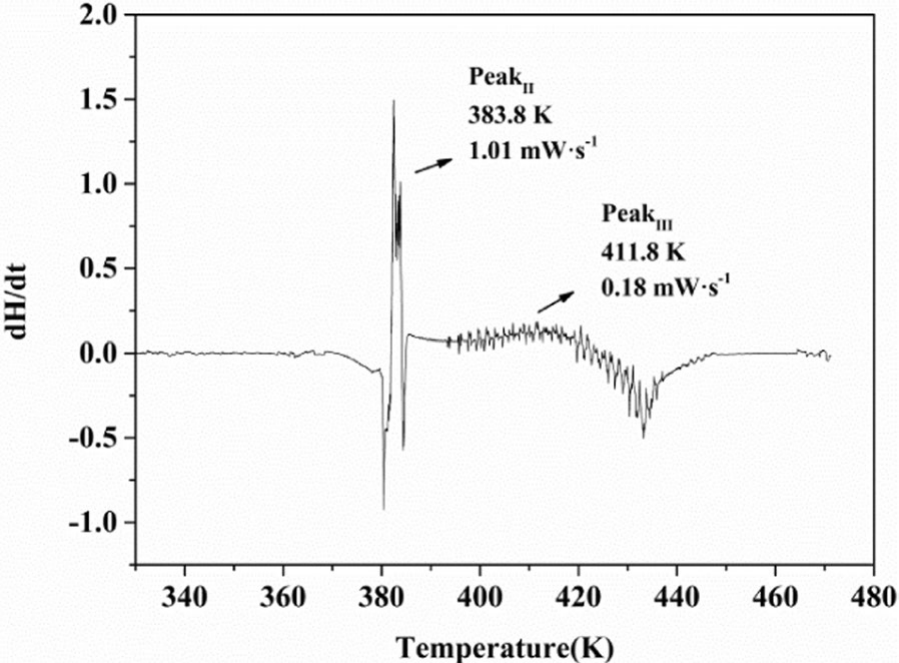
(dH/dt) vs. temperature of BHTOOH

Peak _III_ represents the primary exothermic process of BHTOOH, which includes the initial decomposition temperature (T_i_), the accelerated decomposition temperature (T_a_), the maximum acceleration temperature (T_MEA_), the maximum exothermic temperature (T_max_) and the offset decomposition temperature (T_e_). The first-order derivative of heat with respect to time (dH/dt) was used as the vertical coordinate. Then the temperature was used as the horizontal coordinate to obtain Fig. 11. MEA represents the maximum exothermic rate. Peak_II_ had a significant exothermic rate (MEA = 1.01 mW s^−1^) but gave off little heat (Q_DSC_ = 1.06 J g^−1^). However, Peak_III_ had a smaller exothermic rate (MEA = 0.18 mW s^−1^) but exerted a large amount of heat (Q_DSC_ = 865.0 J g^−1^). According to the UN recommendations on transporting dangerous goods (UNRTDG) [30], BHTOOH should be classified as the fifth type of hazardous substance because its Q_DSC_ exceeds 250.00 J g^−1^. Organic peroxides are hazardous, such as tert-butyl perbenzoate (TBPB), a common organic peroxide initiator in the polymer field, with an exothermic value of about 1279 ± 135 J g^−1^, which makes it very thermally hazardous [31]. If the BHT is oxidized to BHTOOH, heat may be generated during long-term storage and transportation. The initial decomposition temperature and maximum acceleration are related to the decomposition reactivity and the decomposition rate and are essential thermal decomposition parameters. Table 3 lists the main thermal parameters of BHTOOH, which gives a better understanding of its thermal decomposition properties and hazards.

**Table 3:** The main thermal parameters of BHTOOH by DSC

Sample	Absorption or exothermic peak	T_m_ (K)	Q_m_ (J g^−1^)	T_i_ (K)	T_a_ (K)	T_max_ (K)	T_e_ (K)	T_MEA_ (K)	MEA (mW s^−1^)	Q_DSC_ (J g^−1^)
BHTOOH (4.04 mg)	Peak_I_	378.8	−42.62	–	–	–	–	–	–	–
	Peak_II_	–	–	383.7	383.8	384.1	384.9	383.8	1.01	1.06
	Peak_III_	–	–	384.9	391.6	422.6	448.2	411.8	0.18	865.0

### Thermal decomposition characteristics of BHT and BHTOOH in TG

**Fig. 12:**
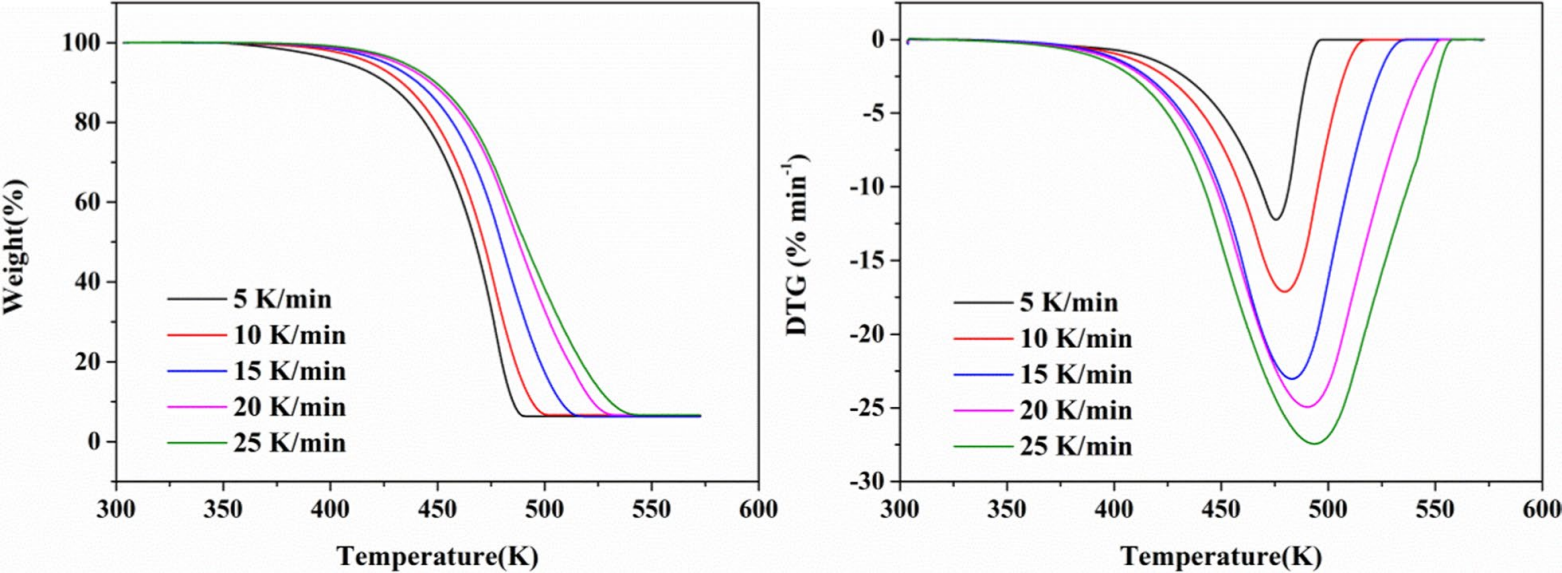
TG and DTG curves for BHT at five different heating rates

**Fig. 13:**
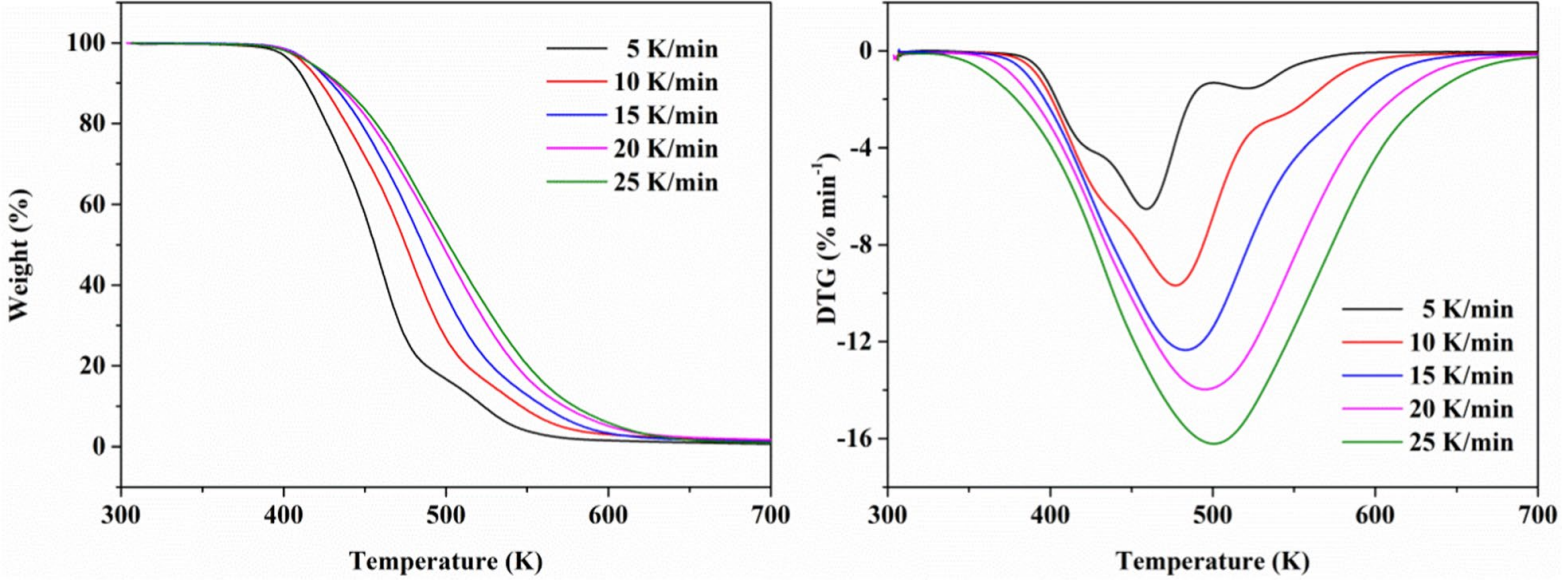
TG and DTG curves for BHTOOH at five different heating rates

To comprehensively consider the hazards of BHTOOH, TG was used for further investigation. The purpose is twofold. One is to derive the point of onset of decomposition, which is an important parameter to measure the hazard of BHTOOH; the other is to examine the reactivity of BHTOOH, the rate of decomposition of which can be observed by the results of TG-DTG [32]. As a comparison, The TG tests of BHT were also performed. The TG and DTG curves of the thermal decomposition reactions of BHT and BHTOOH are shown in Fig. 12 and Fig. 13, respectively.

From the TG curve in Fig. 12, the decomposition region of BHT is in the range of 445.8 K–524.0 K. The peak of the DTG curve is the temperature (T_max_) with the maximum rate of mass change, and T_max_ increases with increasing heating rate. Figure 13 shows that BHTOOH has a clear decomposition zone in 419.1–572.9 K. The weight loss was in the range of 99.09 wt. % (25 K min^−1^)—99.27 wt. % (5 K min^−1^) even when the heating rate conditions were changed. This degradation zone corresponds to the complete decomposition of BHTOOH. There was a tiny reduction in weight loss (< 1 wt. %) in the zone from 306 to 379 K due to the evaporation of water. T_max_ gradually increases from 458.9 K to 500.1 K with an increasing heating rate. The epitaxial onset decomposition temperature (T_i_) is the intersection of the tangent line of the curve’s descending section and the baseline’s extension line. It is an important parameter for understanding the thermal stability of substances. T_e_ represents the temperature at the intersection of the tangent line to the maximum rate of change point of the weight loss descent line and the maximum weight loss line. T_r_ represents the residue temperature, which indicates the temperature at which non-volatile products remain after the volatile products have escaped. To gain more insight into the stability of BHT and BHTOOH, the relevant important thermal decomposition parameters are listed in Table 4.

**Table 4:** The thermal decomposition characteristics of BHT and BHTOOH under different heating conditions

Sample	β (K min^−1^)	T_i_ (K)	T_max_ (K)	T_e_ (K)	T_r_ (K)	Weight loss (%)	The residue (%)
BHT	5	445.8	475.6	484.9	572.9	93.6	6.40
	10	448.6	479.6	491.9	572.5	93.1	6.66
	15	450.3	483.1	504.7	572.3	93.5	6.29
	20	452.8	490.2	519.2	552.5	93.2	6.69
	25	452.4	493.6	523.7	568.2	93.3	6.69
BHTOOH	5	419.1	458.9	492.2	772.4	99.3	0.29
	10	426.0	476.7	522.6	771.7	98.5	1.05
	15	428.3	482.5	543.2	772.0	99.2	0.64
	20	429.8	494.4	564.5	770.3	98.4	1.20
	25	430.5	500.1	572.9	769.8	99.4	0.39

The epitaxial onset decomposition temperature of BHT increased with increasing heating rate from 445.8 K to 452.4 K. The results show that the T_i_ of BHT was greater than that of BHTOOH at different heating rates. This proves that BHT is more stable than BHTOOH. The T_e_ of BHT was lower than BHTOOH’s, indicating that BHT would soon decompose at a specific temperature. In contrast, BHTOOH is more unstable and has more extensive decomposition temperatures. This may be due to the breakage of peroxygen bonds to generate many free radicals, causing the decomposition reaction to occur earlier and become more complex.

### Thermal decomposition kinetics of BHT and BHTOOH by TG

Based on the TG data, the kinetics of the thermal decomposition of BHT and BHTOOH can be calculated. The isotransformation method provides important kinetic parameters and avoids considering decomposition reaction models. To conveniently assess the thermal safety of BHT and BHTOOH, the kinetic parameters at T_max_ were calculated using the method of Kissinger–Akahira–Sunose (KAS) [33], The formula is as follows:

1 $${\text{ln}}\left( {\upbeta /\left( {{\text{T}}^{{2}} } \right)} \right) = {\text{ln}}\left[ {{\text{AR}}/{\text{E}}} \right] - {\text{E}}/\left( {{\text{RT}}} \right)$$

The apparent activation energies of BHT and BHTOOH are listed in Table 5. From Table 5, the apparent activation energies of BHT and BHTOOH were calculated to be 151.8 kJ mol^−1^ and 66.07 kJ mol^−1^, respectively. Activation energy represents the minimum energy required for a chemical reaction to occur. The smaller activation energy implies that the reaction is more likely to occur. The apparent activation energy of BHTOOH was much smaller than that of BHT, which means that BHTOOH was prone to decomposition reactions, and BHT was relatively stable. Industrial safety is of great concern. In the preparation and application of many important chemical products, their decomposition temperatures, activation energies, and exothermic quantities are important thermodynamic parameters [34–39]. BHTOOH can be generated in the industry along with the production and application of BHT, and these data provide a reference for the safety of the BHT-related industries.

**Table 5:** Kinetic parameters obtained by the Kissinger method

Sample	E_α_(kJ mol^−1^)	A(min^−1^)	R^2^
BHT	151.8	2.42 × 10^16^	0.906
BHTOOH	66.1	6.31 × 10^6^	0.985

To further determine the mechanism functions for the thermal decomposition of BHT and BHTOOH, the Malek method [40] was used to determine the most probable mechanism functions (f(α) and g(α)). This method is characterized by being more objective and does not require assumptions.

Combining the reaction rate equation, the Coats-Redfern equation, and the expression for g(α) at a conversion rate of 0.5, the equation for y(α) can be directly derived as follows:

2 $${\text{Y(}}\upalpha ) = \frac{{{\text{f(}}\upalpha {\text{)g(}}\upalpha {)}}}{{{\text{f(0}}{\text{.5)g(0}}{.5)}}} = \frac{{{\text{T}}_{\upalpha }^{2} \left( {\frac{{{\text{d}}\upalpha }}{{{\text{dt}}}}} \right)_{\upalpha } }}{{{\text{T}}_{0.5}^{2} \left( {\frac{{{\text{d}}\upalpha }}{{{\text{dt}}}}} \right)_{0.5} }}$$

**Fig. 14:**
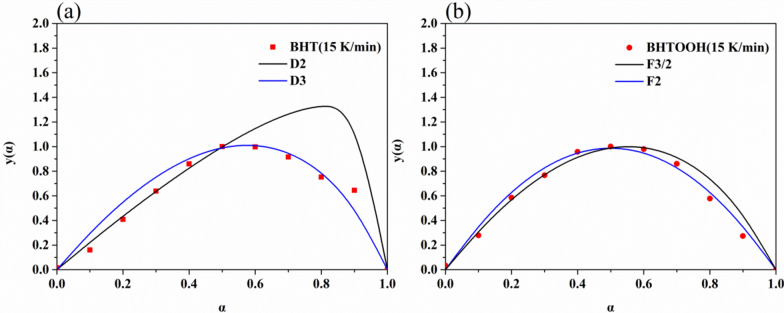
Comparison of theoretical master plots for different mechanism functions and experimental master plot at 15 K min^−1^. **a** BHT; **b** BHTOOH

The 40 common thermal decomposition reaction functions were substituted into y(α) = f(α)g(α)/f(0.5)g(0.5), and the y(α)–α curve was plotted as the standard curve. Then the TG data are substitute into y(α) = (T_α_/T_0.5_)^2^ × (dα/dt)/(dα/dt)_0.5_, and the plotted y(α)–α curve is the experimental curve. Suppose the experimental curve overlaps with the standard curve or the experimental data points all fall on a standard curve. In that case, f(α) or g(α) corresponding to the standard curve is the most probable kinetic mechanism function. The y(α)–α curves of BHT and BHTOOH at 15 K/min and the conformed standard curves are depicted in Fig. 14.

As shown in Fig. 14a, when the reaction process of BHT was 0.2, the temperature was 457.1 K. In Hamama’s study [41], BHT was heated at 458 K for 45 min with evaporation and decomposition, which indicates that gases are produced during the decomposition of BHT. The BHT decomposition reaction progress of 0–0.5 was consistent with the D2 mechanism. This suggests that this process is controlled by two-dimensional diffusion. When the reaction proceeded above 0.5, it conformed to the D3 mechanism; the decomposition process of BHT was changed from one-dimensional diffusion to two-dimensional diffusion, which indicates that the heat transfer in the decomposition has been changed.

For the decomposition of BHTOOH (Fig. 14b), the mechanism differed from that of BHT. At the reaction progress of 0–0.3, the F3/2 chemical reaction kinetic model was followed. The reaction progress of 0.4 changed to the F2 chemical reaction kinetic model. The results show that the thermal decomposition reactions of BHTOOH were complex chemical reactions with a change in reaction level from 3/2 to 2.

### Thermal decomposition products of BHT and BHTOOH

To gain a better understanding of the properties of the thermal decomposition of BHT and BHTOOH, the gaseous products, as well as the liquid products of MCPVT, were identified by GC–MS.

BHT was heated under nitrogen atmosphere and the gaseous products are listed in Table 6. The major decomposition product of BHT was isobutene with a relative content of 51.11%. This indicates that BHT decomposed, stripping off the tert-butyl group to produce isobutene. Table 7 lists the liquid products of thermal decomposition of BHT. 2-tert-butyl 4-methylphenol (3.61%) proved the production of the gaseous product isobutene. In addition, BHT polymerized to the dimer 1,2-Bis(3,5-di-T-butyl-4-hydroxyphenyl)ethane (4.01%).

**Table 6:** Thermal decomposition gas products of BHT identified by GC–MS under a nitrogen atmosphere

No.	Components	Molecular formula	Relative content %	Similarity %
1	Nitrogen	N_2_	36.01	93
2	water	H_2_O	4.18	100
3	Isobutene	C_4_H_8_	51.11	87
4	Ethanol	C_2_H_6_O	8.57	98
5	tert-butanol	C_4_H_10_O	0.13	96

**Table 7:** Thermal decomposition liquid products of BHT identified by GC–MS under a nitrogen atmosphere

No.	Components	Molecular formula	Relative content %	Similarity %
1	2-Tert-Butyl-4-methylphenol	C_11_H_16_O	3.61	97
2	Butylhydroxytoluene	C_15_H_24_O	91.16	95
3	3,5-Di-tert-butyl-4-hydroxybenzaldehyde	C_15_H_22_O_2_	0.38	83
4	1,2-Bis(3,5-di-T-butyl-4-hydroxyphenyl)ethane	C_30_H_46_O_2_	4.01	94
5	Unidentified product	–	0.83	–

The gas products of BHTOOH are listed in Table 8. The thermal decomposition products of BHTOOH under different gas atmospheres were broadly similar. The main gaseous product was isobutene, with a relative content of 27.99% in nitrogen, 27.16% in oxygen, and 17.62% in air. In the case of nitrogen, the additional product is isobutyraldehyde, with a relative content of 0.09%.

**Table 8:** Thermal decomposition gas products of BHTOOH identified by GC–MS under three gaseous atmospheres

No.	Components	Molecular formula	Relative content %				Similarity %
			N_2_	O_2_	Air^a^	Air^b^	
1	Oxygen	O_2_	0.18	58.2	54.4	57.4	92
2	Nitrogen	N_2_	60.7	–	–	–	93
3	Water	H_2_O	8.30	3.88	4.48	4.98	100
4	Methyl formate	C_2_H_4_O_2_	1.29	1.04	0.51	0.58	99
5	Acetone	C_3_H_6_O	0.34	8.32	15.8	18.7	98
6	Ethyl formate	C_3_H_6_O_2_	0.07	0.74	1.12		92
7	Isobutylene	C_4_H_8_	28.0	27.2	23.4	17.6	97
8	tert-Butanol	C_4_H_10_O	0.73	0.40	0.35	0.55	97
9	Isobutyraldehyde	C_4_H_8_O	0.09	–	–	–	92
10	Pivalaldehyde	C_5_H_10_O	0.27	0.23	0.06	0.23	93

^a^No gas filling

^b^P_0_ = 0.4 MPa

The liquid products of BHTOOH are shown in Table 9. Under the different gas atmospheres, the products were approximately the same, with different relative contents. BHTOOH underwent thermal decomposition in all three gas atmospheres, yielding similar products. The major liquid products were V, IX, XI and XIII. V (BHT) is the predominant product of BHTOOH decomposition. XIII is one of the toxic metabolites of BHT. Once BHT is oxidized, toxic products can be ingested into the human body and pose a threat to human health. The polymerization products were XI and XVII.

**Table 9:** Thermal decomposition liquid products of BHTOOH identified by GC–MS under three gaseous atmospheres

No.	Components	Molecular formula	Relative content %				Similarity %
			N_2_	O_2_	Air^a^	Air^b^	
1	2,6-Bis(1,1-dimethylethyl)-4,4-dimethyl-2,5-cyclohexadien-1-one (I)	C_16_H_26_O	0.76	1.10	0.63	1.18	85
2	2,4-Dimethyl-6-tert-butylphenol (II)	C_12_H_18_O	0.73	0.88	0.75	0.87	93
3	2,2-Dimethyl-5-(1-methylpropenyl)tetrahydrofuran (III)	C_10_H_18_O	1.70	2.00	1.57	2.36	93
4	2,6-Di-tert-butyl-1,4-benzoquinone (IV)	C_14_H_20_O_2_	1.16	5.48	0.85	2.12	88
5	2,6-Di-tert-butyl-4-methylphenol (V)	C_15_H_24_O	38.0	21.2	34.8	38.3	95
6	1,2,3-Trimethoxybenzene (VI)	C_9_H_12_O_3_	1.81	–	–	–	80
7	3,4-Dimethoxyacetophenone (VII)	C_10_H_12_O_3_	1.1	0.94	1.04	1.10	87
8	1,3,5-Tri-tert-butyl-3-(3,5-di-tert-butyl-4-hydroxybenzyl)bicyclo[4.1.0]hept-4-en-2-one (VIII)	C_10_H_12_O_4_	1.54	2.61	1.38	1.89	84
9	2,6-Di-tert-butyl-4-ethylphenol (IX)	C_16_H_26_O	2.75	1.85	2.53	3.24	95
10	2,4,6-Tris(1,1-dimethylethyl)-4-methyl-2,5-cyclohexadien-1-one (X)	C_19_H_32_O	–	–	–	0.56	89
11	Bicyclo[4.1.0]hept-4-en-2-one,3-[[3,5-bis(1,1-dimethylethyl)-4-hydroxyphenyl]met-hyl]-1,3,5-tris(1,1-dimethylethyl) (XI)	C_34_H_54_O_2_	3.80	4.71	3.82	4.50	83
12	2,5-Di-tert-butylhydroquinone (XII)	C_14_H_22_O_2_	1.25	–	1.34	–	83
13	3,5-Di-tert-butyl-4-hydroxybenzaldehyde (XIII)	C_15_H_22_O_2_	4.66	12.7	6.35	6.55	95
14	Methyl 3,5-bis(1,1-dimethylethyl)-4-hydr-oxy benzoate (XIV)	C_16_H_24_O_3_	–	–	–	1.9	84
15	3′,5′-Di-tert-butyl-4′-hydroxyacetophenone (XV)	C_16_H_24_O_2_	–	1.70	–	–	95
16	3,5-Di-tert-butyl-4-hydroxybenzoic acid (XVI)	C_15_H_22_O_3_	–	1.63	–	–	88
17	4,4′-(1,2-Ethanediyl)bis[2,6-bis(1,1-dimeth-ylethyl)phenol (XVII)	C_30_H_46_O_2_	16.3	–	19.7	–	90
18	Unidentified product	–	29.1	43.7	26.6	34.1	–

^a^No gas filling

^b^Fill with 0.4 MPa air

Under an oxygen atmosphere, there was a greater variety of products. The production of XV and XVI indicates that the products after the decomposition of BHTOOH will continue to react with oxygen to produce different oxidation products. With more oxygen, a more significant proportion of oxidation products were produced.

Even under a nitrogen atmosphere, BHTOOH was decomposed. The reconstituted products dominated the decomposed products, with a smaller proportion of oxidation products than under the other two atmospheres.

In air, BHTOOH was also decomposed after heating. The pressure increased, and the proportion of products increased accordingly. It can be seen from the products that the decomposition of BHTOOH is a free radical reaction process. BHTOOH was first decomposed, generating many free radicals, and then the free radicals combined or initiated products to continue the reaction.

### Thermal decomposition pathway of BHTOOH

**Scheme 1:**
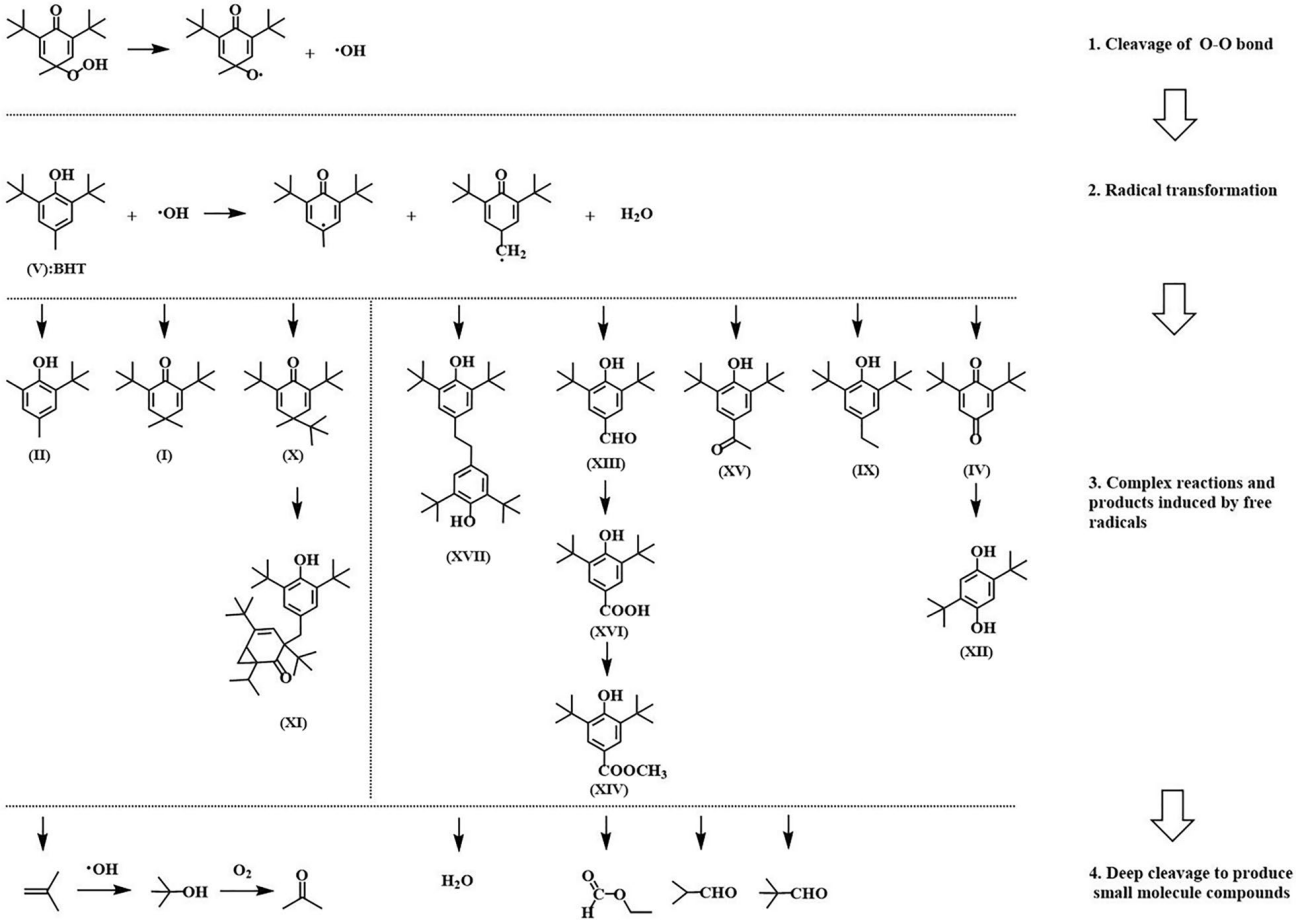
Possible thermal decomposition pathways of BHTOOH

The thermal decomposition of BHTOOH was a complex process, as evidenced by the variety of products. The analysis of the thermal decomposition products helps understand the instability of BHTOOH and the pathways of thermal decomposition. Possible reaction pathways are shown in Scheme 1.

Organic peroxides are extremely unstable and prone to decomposition due to their peroxide bonds [42]. When BHTOOH was heated under different gas atmospheres, BHTOOH first broke the O–O bond to form BHTO· and ·OH radicals. BHTO· can be oxidized to IV [20].

The main product of BHTOOH decomposition was BHT (V). Because thermal decomposition generated many free radicals, BHT was triggered by free radicals to undergo a series of reactions. Free radicals initiated BHT to produce cyclohexadienone structures with radicals where electrons can be transferred to the ring or the p-methyl group. This result was consistent with the metabolic process of BHT, which had two main metabolic processes: the oxidation of alkyl substituents and the oxidation of aromatic ring systems. The metabolites and the BHTOOH thermal decomposition products were also similar [43]. The radical on the ring combined with methyl to create I and with tert-butyl to form X. It may also lose the tert-butyl group and combine with methyl to form II. Similarly, the radicals on the methyl group can combine to form a dimer of BHT (XVII) [44]. The system contained large amounts of ·OH, ·CH_3_, and O_2_, which the benzyl group combined with and oxidized to form oxidation products such as XIII and XV [45]. Of these products, XIII, IV, and XVI are common toxic metabolites. XIII is toxic to the heart and is a potential teratogen in aquatic organisms [7]. XVI is the main metabolite of BHT and IV has been found to cause DNA strand damage [46]. This toxic substance can be produced in the decomposition of BHTOOH, and the presence of BHTOOH in BHT-related products increases the risk of toxic products entering the human body through the food chain.

In addition, p-benzoquinone can be reduced by ·H and rearranged to form XII. A large amount of gas was generated when entering the deep oxidation stage. This was reflected in the MCPVT results as a rapid increase in pressure. The formation of gas products was complex, mostly from free radical reactions and further oxidation. For example, isobutylene was formed from tert-butyl by a free radical reaction. Isobutylene combined with ·OH to form tert-butyl alcohol, which was further oxidized to acetone [28].

In conclusion, the thermal decomposition of BHTOOH was a very complex reaction, and the whole process was divided into four steps: (1) the O–O bond was broken, generating many radicals; (2) ·OH initiated BHT and formed two kinds of radicals; (3) the BHT radicals initiated a complex reaction and generated a large number of products; (4) small molecule compounds was generated by the deep cleavage.

## Conclusions

The thermal decomposition properties of BHT and BHTOOH were studied using MCPVT. The thermal hazard and the kinetics of thermal decomposition were studied by DSC and TG. The relevant thermodynamic parameters were obtained. This study provides a better understanding of the peroxides of BHT. As a commonly used food additive, the stability of BHT and the thermal hazards of its peroxide BHTOOH are of interest. The following conclusions were obtained:

1. The results of MCPVT show that BHT was thermally stable with no significant change in temperature and pressure below 400 K under a nitrogen atmosphere. The peroxide of BHT, BHTOOH, is thermally unstable compared to BHT. BHTOOH underwent thermal decomposition in the presence of nitrogen, oxygen, air, and no gas filling. The T–t and P–t curves imply a vigorous exothermic reaction of BHTOOH under a nitrogen atmosphere. The initial exothermic temperature was 375.2 K, and the pressure increased rapidly (ΔP = 0.0731 MPa). Even without gas filling, thermal decomposition of BHTOOH occurred with an initial exothermic temperature of 370.5 K.
2. DSC was used to study the thermal hazards of BHT and BHTOOH. The DSC curve reveals that BHT had no exothermic reaction. BHTOOH decomposed immediately after melting. The primary decomposition started at 384.9 K with an exotherm of 865.0 J g^−1^. The results show that although BHT is relatively stable, it is potentially harmful once oxidized to peroxide BHTOOH.
3. The thermal weight loss curves of BHT and BHTOOH were plotted by the TG test. The TG and DTG curves showed that the epitaxial onset decomposition temperature of BHT was higher than that of BHTOOH at the same heating rates. The activation energy of the thermal decomposition of BHT and BHTOOH was calculated using the Kissinger method. Their activation energies were 151.8 kJ mol^−1^ and 66.07 kJ mol^−1^, respectively. The results indicate that BHT was not prone to decomposition, and BHTOOH was very unstable and prone to decomposition. The most probable kinetic mechanisms were discussed for BHT and BHTOOH, both of which have altered decomposition processes that cannot be described by a single kinetic model. BHT was transformed from two-dimensional diffusion to three-dimensional diffusion. The decomposition of BHTOOH was a complex chemical reaction, and the reaction level changed from 1.5 to 2.
4. The gaseous and liquid products of the thermal decomposition of BHT and BHTOOH were detected by GC–MS. The main gaseous products of BHT were decomposition products such as isobutene. Polymerization also occurred, producing dimers of BHT. Similar products of BHTOOH were detected under different gaseous atmospheres, with more oxidation products under an oxygen atmosphere. BHTOOH thermal decomposition products contain toxic metabolites. The pathway of thermal decomposition of BHTOOH was postulated. In summary, the O–O bond of BHTOOH is first broken, generating many free radicals, which then trigger its cleavage and reorganization, accompanied by an oxidation reaction.

In conclusion, this study investigates the thermal decomposition properties of BHT and BHTOOH, which can help to provide a valid reference value for BHT applications in different scenarios. BHT is safe to use at ambient temperature and pressure but becomes dangerous once it oxidizes to BHTOOH, which decomposes to give off large amounts of heat and produces organic gases as well as toxic products. Understanding the thermal decomposition properties of BHTOOH can prevent accidents during the generation, use and transportation of BHT.

## Data Availability

The datasets used and/or analyzed during the current study are available from the corresponding author on reasonable request.
